# The association between BRCA1 gene polymorphism and cancer risk: a meta-analysis

**DOI:** 10.18632/oncotarget.24064

**Published:** 2018-01-06

**Authors:** Gui-Ping Xu, Qing Zhao, Ding Wang, Wen-Yue Xie, Li-Jun Zhang, Hua Zhou, Shi-Zhi Chen, Li-Fang Wu

**Affiliations:** ^1^ Transfusion Department, The Second Affiliated Hospital of Chongqing Medical University, Chongqing, China; ^2^ Department of Laboratory Medicine, The Second Affiliated Hospital of Chongqing Medical University, Chongqing, China; ^3^ Department of Oncology, The Second Affiliated Hospital of Chongqing Medical University, Chongqing, China

**Keywords:** BRCA1, polymorphism, meta-analysis, cancer

## Abstract

Many studies have reported that BRCA1 polymorphisms are associated with cancer risk, but the results remain controversial. The purpose of this meta-analysis is to evaluate the relationship between BRCA1 polymorphisms (rs799917, rs1799950, rs1799966, or rs16941) and cancer risk. Relevant studies were identified via a systematic search of the PubMed, Embase, and Web of Science databases up to July 31, 2017. Odds ratios (ORs) with 95% confidence intervals (CIs) were calculated to examine the strength of the associations. Thirty-five studies published in 19 publications involving 28,094 cases and 50,657 controls were included in this meta-analysis. There was no obvious association between rs799917, rs1799966, or rs16941 polymorphisms and overall cancer risk in any genetic models. However, subgroup analyses revealed that the rs799917 polymorphism could decrease the risk of cervical cancer, esophageal squamous cell carcinoma (ESCC), gastric cancer, and non-Hodgkin lymphoma (NHL) among Asian populations in one or more genetic models and that rs16941 could increase overall cancer risk among Caucasian populations in the homozygote and recessive models. Our meta-analysis also indicated that rs1799950 could decrease the breast cancer (BC) risk among Caucasian populations in the homozygote and recessive models. In summary, our results suggest that BRCA1 polymorphisms may play an important role in the etiology of cancer. However, due to the limited number of studies, these findings should be confirmed by new studies with larger sample sizes that address various types of cancer.

## INTRODUCTION

Year by year, cancer incidence and mortality are increasing worldwide, seriously affecting human health and generating a huge economic burden. Cancer is thought to be the result of interactions between genes and environmental factors [1], such as tobacco use, alcohol intake, overweight and infection [2]. Single-nucleotide polymorphisms (SNPs) are the most common genetic alterations between individuals, and it is reported that SNPs in DNA repair genes have a relationship with a variety of cancers [3].

*BRCA1* is a tumor suppressor gene located on chromosome 17q21. It was mapped in 1990 and cloned in 1994 [4, 5]. The *BRCA1* gene was first identified as a strong candidate gene influencing susceptibility to breast and ovarian cancer [5]. BRCA1 is comprised of multiple functional domains and interacts with many proteins, including tumor suppressors, oncogenes, DNA damage repair proteins, cell cycle regulators, and transcriptional activators and repressors [6, 7]. BRCA1 deficiency can lead to defects in the S phase, G2/M phase, and spindle checkpoints, causing genetic instability and thus triggering DNA damage response, further increasing the risk of tumor formation [8–10]. It is reported that BRCA1 is expressed in many tissues, such as the lymph nodes, skin, bladder, cervix, liver, uterus, prostate, pancreas, lung, kidney, bone, brain, and is related to various types of cancer, including breast, ovarian, endometrial, pancreatic, prostate, and colon cancers [11–13].

Many studies have been conducted to examine the association between *BRCA1* polymorphisms and the risk of various cancers, including breast cancer, ovarian cancer (OC), cervical cancer, ESCC, gastric cancer, chronic myeloid leukemia (CML), and NHL [14–32]; however, the results remain inconsistent or even contradictory. For example, Dunning et al. found that rs799917 showed no significant association with breast cancer [14], while Nicoloso et al. suggested that rs799917 increased the risk of breast cancer [23]. Studies regarding rs1799966, rs1799950, and rs16941 are similarly inconsistent, so we performed a meta-analysis to more precisely determine the association between *BRCA1* polymorphisms and cancer risk.

## RESULTS

### Characteristics of the studies

**Figure 1:**
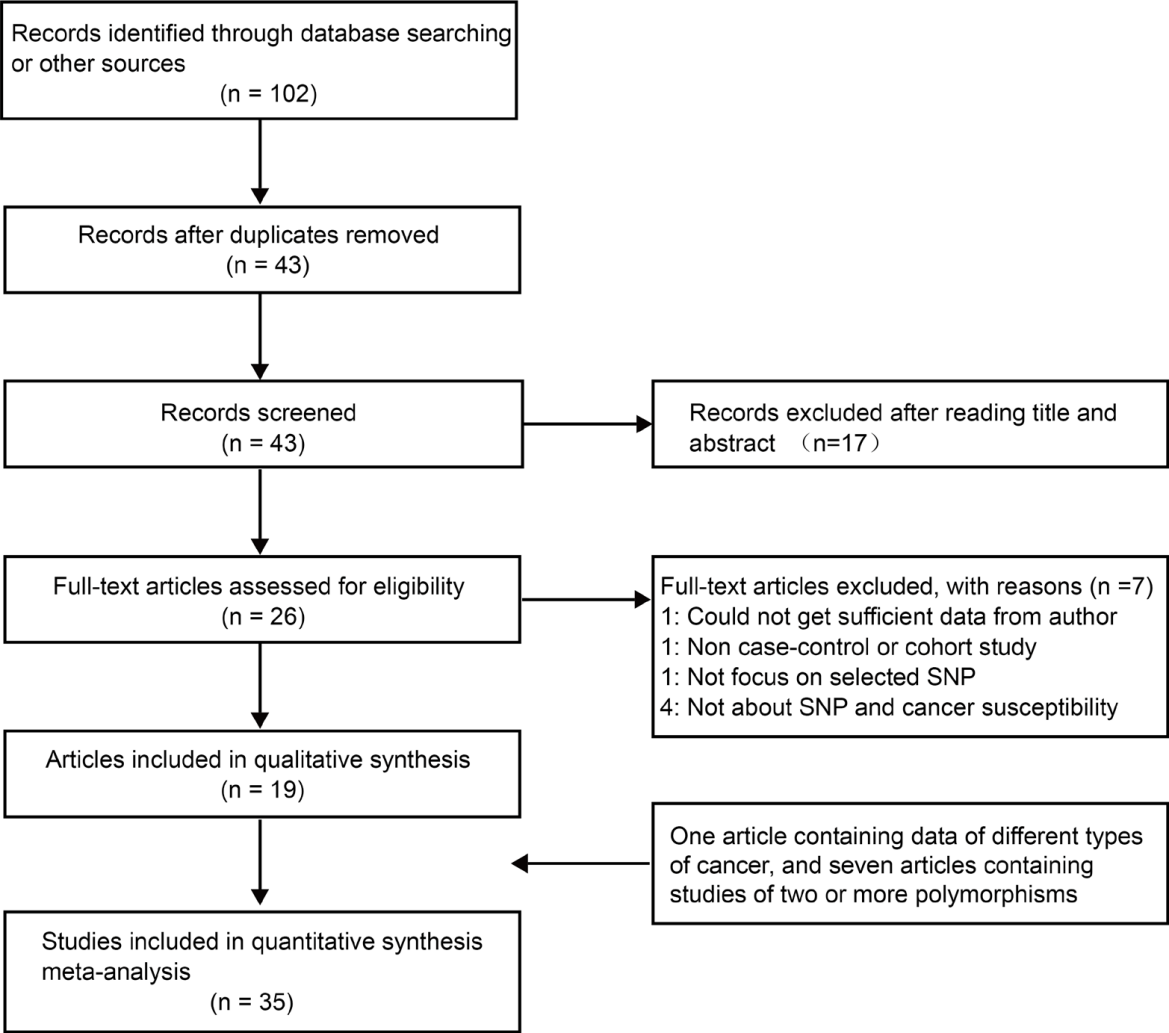
The flow diagram of included/excluded studies

The flow diagram of the study selection process is shown in Figure 1. A total of 102 articles were obtained from a database search and from other sources. After duplicate removal and subsequent title and abstract assessment, 26 articles were left for further full-text review. Based on the inclusion and exclusion criteria, 19 articles remained [14–32]. One article containing data regarding various types of cancer was treated as independent studies [14], and there were seven articles containing studies of various *BRCA1* polymorphisms [14, 16, 17, 21, 22, 24, 27]. Altogether, 35 studies contained in 19 articles involving 28,094 cases and 50,657 controls were included in this meta-analysis. The main characteristics of the included studies are summarized in Table 1. These articles were published from 1997 to 2016. Among them, ten articles were carried out in Caucasian populations, six in Asian populations, and three in other populations. The quality of the studies was evaluated based on a quality assessment scale (Supplementary Table 1). The distributions of genotypes and allele frequencies in the cases and controls are shown in Table 2.

**Table 1:** Characteristics of the studies included in the meta-analysis

First author	Year	Country	Ethnicity	Cancer type	Genotyping method	Control source
Dunning [14]	1997	UK	Caucasian	Breast cancer Ovarian cancer	ASOs hybridisation	PB
Baynes [15]	2007	UK	Caucasian (＞ 98%)	Breast cancer	Taqman	PB
Soucek [16]	2007	Czech Republic	Caucasian	Breast cancer	PCR-RFLP	HB
Chang [17]	2008	USA	Caucasian	glioblastoma	ParAllele SNP panel	PB
Wang [18]	2009	China	Asian	Breast cancer	PCR-PIRA	PB
Huo [19]	2009	China	Asian	Breast cancer	PCR-PIRA	PB
Zhou [20]	2009	China	Asian	Cervical cancer	PCR-PIRA	PB
Dombernowsky [21]	2009	Denmark	Caucasian	Breast cancer	Taqman	PB
Abbas [22]	2010	Germany	Caucasian	Breast cancer	MALDI-TOF MS	PB
Nicoloso [23]	2010	USA	Caucasian	Breast cancer	BigDye Terminator Reaction	NR
Xu [24]	2012	USA	Mix	SGC	PCR-RFLP	HB
Zhang [25]	2013	China	Asian	ESCC	PCR-RFLP	PB
Ricks-Santi [26]	2013	USA	Caucasian	Breast cancer	TaqMan	PB
Wu [27]	2013	USA	Caucasian	Breast cancer	Taqman	FB
Hasan [28]	2013	Saudi Arabia	Arab	Breast cancer	Taqman	HB
Kim [29]	2014	Korea	Asian	NHL	PCR-PIRA	PB
Wójcicka [30]	2014	Poland	Caucasian	PTC	iPLEX Gold system	PB
Wang [31]	2015	China	Asian	Gastric cancer	PCR-RFLP	PB
Gutierrez [32]	2016	Mexico	Latin American	CML	Taqman	Blood bank

SGC: Salivary Gland Carcinoma; ESCC: esophageal squamous cell carcinoma; NHL: Non-Hodgkin Lymphoma; PTC: Papillary Thyroid Carcinoma; CML: chronic myeloid leukemia; ASOs: allele-specific oligonucleotides; PCR-PIRA: PCR primer introduced restriction analysis assay; PCR-RFLP: PCR restriction fragment length polymorphism; MALDI-TOF MS: matrix-assisted laser desorption/ionization time-of-flight mass spectrometry; PB: population-based; HB: hospital-based; FB: family-based; NR: no record.

**Table 2:** *BRCA1* polymorphisms genotype distribution and allele frequency in cases and controls

	Genotype (*N*)								Allele frequency (*N*)				HWE	Score
	Case				Control				Case		Control			
rs799917	Total	CC	CT	TT	Total	CC	CT	TT	C	T	C	T		
Dunning [14](BC)	801	342	370	89	572	266	250	56	1054	548	782	362	0.805	13
Dunning [14](OC)	223	102	94	27	572	266	250	56	298	148	782	362	0.805	12
Chang [17]	112	51	51	10	112	55	38	19	153	71	148	76	0.010	9
Wang [18]	1004	381	483	140	1008	403	463	142	1245	763	1269	747	0.626	14
Huo [19]	568	215	283	70	624	255	285	84	713	423	795	453	0.757	14
Zhou [20]	404	166	196	42	404	158	183	63	528	280	499	309	0.410	13
Dombernowsky [21]	1201	550	496	155	4120	1756	1896	467	1596	806	5408	2830	0.187	13
Abbas [22]	3136	1403	1377	356	5470	2433	2396	641	4183	2089	7262	3678	0.168	13
Nicoloso [23]	247	90	118	39	185	90	75	20	298	196	255	115	0.465	7
Xu [24]	156	71	62	23	511	198	226	87	204	108	622	400	0.105	11
Zhang [25]	1128	482	530	116	1150	444	524	182	1494	762	1412	888	0.188	13
Wu [27]	335	108	164	63	408	120	211	77	380	290	451	365	0.354	12
Hasan [28]	100	31	37	32	100	30	36	34	99	101	96	104	0.005	7
Kim [29]	687	364	273	50	1700	828	715	157	1001	373	2371	1029	0.882	14
Wang [31]	660	286	313	61	800	302	365	133	885	435	969	631	0.204	13
Gutierrez [32]	312	147	129	36	469	200	210	59	423	201	610	328	0.737	12
rs1799950	Total	AA	AG	GG	Total	AA	AG	GG	A	G	A	G		
Dunning [14] (BC)	765	684	81	0	631	550	74	7	1449	81	1174	88	0.016	10
Dunning [14] (OC)	230	195	35	0	631	550	74	7	425	35	1174	88	0.016	9
Baynes [15]	2182	1955	221	6	2273	2004	256	13	4131	233	4264	282	0.125	15
Soucek [16]	305	261	43	1	305	243	56	6	565	45	542	68	0.201	11
Dombernowsky [21]	1200	1048	147	5	4119	3589	513	17	2243	157	7691	547	0.771	13
Abbas[22]	3139	2711	417	11	5481	4762	679	40	5839	439	10203	759	0.004	10
Xu[24]	156	132	24	0	511	455	56	0	288	24	966	56	0.190	11
rs1799966	Total	AA	AG	GG	Total	AA	AG	GG	A	G	A	G		
Soucek[16]	449	270	146	33	295	127	132	36	686	212	386	204	0.851	11
Chang[17]	111	53	49	9	112	56	39	17	155	67	151	73	0.028	9
Dombernowsky [21]	1198	557	508	133	4119	1850	1834	435	1622	774	5534	2704	0.535	13
Abbas [22]	3140	1422	1365	353	5487	2445	2391	651	4209	2071	7281	3693	0.073	13
Xu [24]	156	80	62	14	511	229	233	49	222	90	691	331	0.353	11
Wu [27]	317	132	143	42	386	162	182	42	407	227	506	266	0.388	12
rs16941	Total	AA	AG	GG	Total	AA	AG	GG	A	G	A	G		
Soucek [16]	305	130	142	33	305	138	131	36	402	208	407	203	0.567	11
Chang [17]	110	51	48	11	109	55	36	18	150	70	146	72	0.008	9
Dombernowsky [21]	1199	563	491	145	4120	1854	1835	431	1617	781	5543	2697	0.463	13
Xu [24]	156	81	61	14	511	230	227	54	223	89	687	335	0.856	11
Ricks-Santi [26]	267	121	124	22	525	255	227	43	366	168	737	313	0.446	12
Wójcicka [30]	1635	807	667	161	2021	1074	792	155	2281	989	2940	1102	0.592	14

HWE: Hardy-Weinberg equilibrium.

### Meta-analysis of rs799917

We assessed the association between the *BRCA1* rs799917 polymorphism and cancer risk by calculating the ORs and their 95% CI using the following five genetic models: the allele model (T vs. C), the homozygote model (TT vs. CC), the heterozygote model (CT vs. CC), the dominant model (TT+CT vs. CC), and the recessive model (TT vs. CT+CC). The main results of the meta-analysis for rs799917 polymorphism are shown in Table 3. In total, there were sixteen studies with 11,074 cases and 18,205 controls for rs799917 polymorphism. In the overall analysis, no significant association was observed between rs799917 polymorphism and cancer risk in any genetic models (Table 3).

**Table 3:** Meta-analysis of the association between rs799917 polymorphism and cancer risk

Subgroup	No.	T vs. C			TT vs. CC			CT vs. CC			TT+CT vs. CC			TT vs. CT+CC		
		OR (95%Cl)	POR	Ph	OR (95%Cl)	POR	Ph	OR (95%Cl)	POR	Ph	OR (95%Cl)	POR	Ph	OR (95%Cl)	POR	Ph
Overall	16	0.95 (0.89–1.01)^∗^	0.106	0.001	0.87 (0.75–1.02)^∗^	0.080	< 0.001	0.98 (0.91–1.05)^∗^	0.533	0.078	0.96 (0.89–1.03)^∗^	0.241	0.022	0.88 (0.77–1.01)^∗^	0.078	<0.001
Ethnicity																
Caucasian	7	1.00 (0.96–1.05)	0.901	0.111	1.03 (0.93–1.14)	0.608	0.187	1.02 (0.89–1.17)^∗^	0.801	0.023	1.03 (0.91–1.16)^∗^	0.680	0.041	1.04 (0.94–1.15)	0.419	0.196
Asian	6	0.89 (0.80–0.99)^∗^	0.032	0.006	0.72 (0.56–0.93)^∗^	0.011	0.003	0.98 (0.90–1.07)	0.653	0.291	0.92 (0.82–1.04)^∗^	0.194	0.064	0.72 (0.58–0.89)^∗^	0.003	0.013
Others	3	0.87 (0.75–1.02)	0.078	0.842	0.81 (0.59–1.11)	0.189	0.887	0.83 (0.66–1.04)	0.101	0.802	0.82 (0.67–1.01)	0.067	0.802	0.89 (0.66–1.18)	0.410	0.971
Cancer type																
Breast cancer	8	1.01 (0.97–1.05)	0.716	0.171	1.03 (0.93–1.13)	0.607	0.484	1.03 (0.92–1.16)^∗^	0.571	0.023	1.04 (0.93–1.15)^∗^	0.484	0.037	1.03 (0.94–1.12)	0.592	0.607
(Caucasian)	5	1.03 (0.94–1.13)^∗^	0.522	0.045	1.03 (0.92–1.15)	0.604	0.176	1.01 (0.86–1.18)^∗^	0.951	0.012	1.03 (0.89–1.19)^∗^	0.732	0.013	1.05 (0.95–1.16)	0.383	0.340
(Asian)	2	1.04 (0.94–1.15)	0.436	1.000	1.02 (0.82–1.27)	0.838	0.818	1.13 (0.97–1.31)	0.108	0.680	1.11 (0.96–1.28)	0.165	0.785	0.96 (0.78–1.17)	0.672	0.678
(Arab)	1	0.94 (0.64–1.39)	0.764	----	0.92 (0.45–1.83)	0.793	----	1.00 (0.50–1.96)	0.988	----	0.95 (0.52–1.74)	0.878	----	0.91 (0.51–1.65)	0.764	----
Other cancers																
(Asian)																
Cervical cancer	1	0.86 (0.70–1.05)	0.134	----	0.64 (0.41–0.99)	0.046	----	1.02 (0.76–1.37)	0.899	----	0.92 (0.70–1.22)	0.566	----	0.63 (0.41–0.95)	0.029	----
ESCC	1	0.81 (0.72–0.92)	0.001	----	0.59 (0.45–0.77)	<0.001	----	0.93 (0.78–1.11)	0.433	----	0.84 (0.71–1.00)	0.045	----	0.61 (0.48–0.78)	< 0.001	----
Gastric cancer	1	0.76 (0.65–0.88)	<0.001	----	0.48 (0.34–0.68)	<0.001	----	0.91 (0.73–1.13)	0.379	----	0.79 (0.64–0.98)	0.030	----	0.51 (0.37–0.71)	< 0.001	----
NHL	1	0.86 (0.75–0.99)	0.032	----	0.72 (0.52–1.02)	0.064	----	0.87 (0.72–1.05)	0.138	----	0.84 (0.71–1.01)	0.059	----	0.77 (0.55–1.08)	0.125	----
(Caucasian)																
Ovarian cancer	1	1.07 (0.85–1.36)	0.554	----	1.26 (0.75–2.10)	0.381	----	0.98 (0.71–1.36)	0.907	----	1.03 (0.76–1.41)	0.846	----	1.27 (0.78–2.07)	0.338	----
Glioblastoma	1	0.90 (0.61–1.34)	0.615	----	0.57 (0.24–1.34)	0.194	----	1.45 (0.82–2.55)	0.201	----	1.15 (0.68–1.95)	0.593	----	0.48 (0.21–1.09)	0.078	----
(other ethnicities)																
SGC	1	0.82 (0.63–1.07)	0.150	----	0.74 (0.43–1.26)	0.263	----	0.77 (0.52–1.13)	0.179	----	0.76 (0.53–1.09)	0.132	----	0.84 (0.51–1.39)	0.502	----
CML	1	0.88 (0.71–1.10)	0.260	----	0.83 (0.52–1.32)	0.434	----	0.84 (0.62–1.14)	0.250	----	0.84 (0.63–1.11)	0.218	----	0.91 (0.58–1.41)	0.663	----
Control source																
PB	11	0.94 (0.88–1.01)^∗^	0.089	0.002	0.85 (0.71–1.01)^∗^	0.065	<0.001	0.97 (0.92–1.03)	0.343	0.126	0.96 (0.89–1.03)^∗^	0.236	0.064	0.84 (0.71–1.00)^∗^	0.056	< 0.001
HB	2	0.86 (0.69–1.07)	0.172	0.578	0.80 (0.52–1.21)	0.289	0.637	0.82 (0.58–1.15)	0.240	0.512	0.81 (0.59–1.10)	0.172	0.520	0.87 (0.60–1.27)	0.477	0.838
others	3	1.05 (0.80–1.38)^∗^	0.725	0.016	1.09 (0.68–1.74)^∗^	0.720	0.070	1.02 (0.71–1.47)^∗^	0.913	0.035	1.05 (0.71–1.55)^∗^	0.824	0.013	1.05 (0.82–1.36)	0.681	0.326
Quality score																
≥12	12	0.94 (0.88–1.00)^∗^	0.054	0.003	0.86 (0.73–1.01)^∗^	0.063	<0.001	0.96 (0.91–1.02)	0.166	0.202	0.94 (0.88–1.01)^∗^	0.099	0.081	0.87 (0.75–1.02)^∗^	0.085	<0.001
< 12	4	1.01 (0.76–1.35)^∗^	0.930	0.028	0.96 (0.57–1.61)^∗^	0.864	0.057	1.14 (0.78–1.68)^∗^	0.496	0.066	1.09 (0.74–1.60)^∗^	0.672	0.036	0.94 (0.70–1.25)	0.662	0.129

SGC: Salivary Gland Carcinoma; ESCC: esophageal squamous cell carcinoma; NHL: Non-Hodgkin Lymphoma; PTC: Papillary Thyroid Carcinoma; CML: chronic myeloid leukemia; OR, odds ratio; 95% CI, 95% confidence interval; *P*OR, pool *P* value; *P*h, *P* value of heterogeneity test; ^*^indicates that the OR, 95% Cl, and corresponding *P*OR were calculated based on the random-effects model; otherwise, the fixed-effects model was used.

In the subgroup analyses by ethnicity, we observe a significant association between rs799917 polymorphism and cancer risk among Asian populations in the allele, homozygote, and recessive models (T vs. C: OR, 0.89, 95% Cl, 0.80–0.99, *P* = 0.032; TT vs. CC: OR, 0.72, 95% Cl, 0.56–0.93, *P* = 0.011; TT vs. CT + CC: OR, 0.72, 95% Cl, 0.58–0.89, *P* = 0.003).

**Figure 2:**
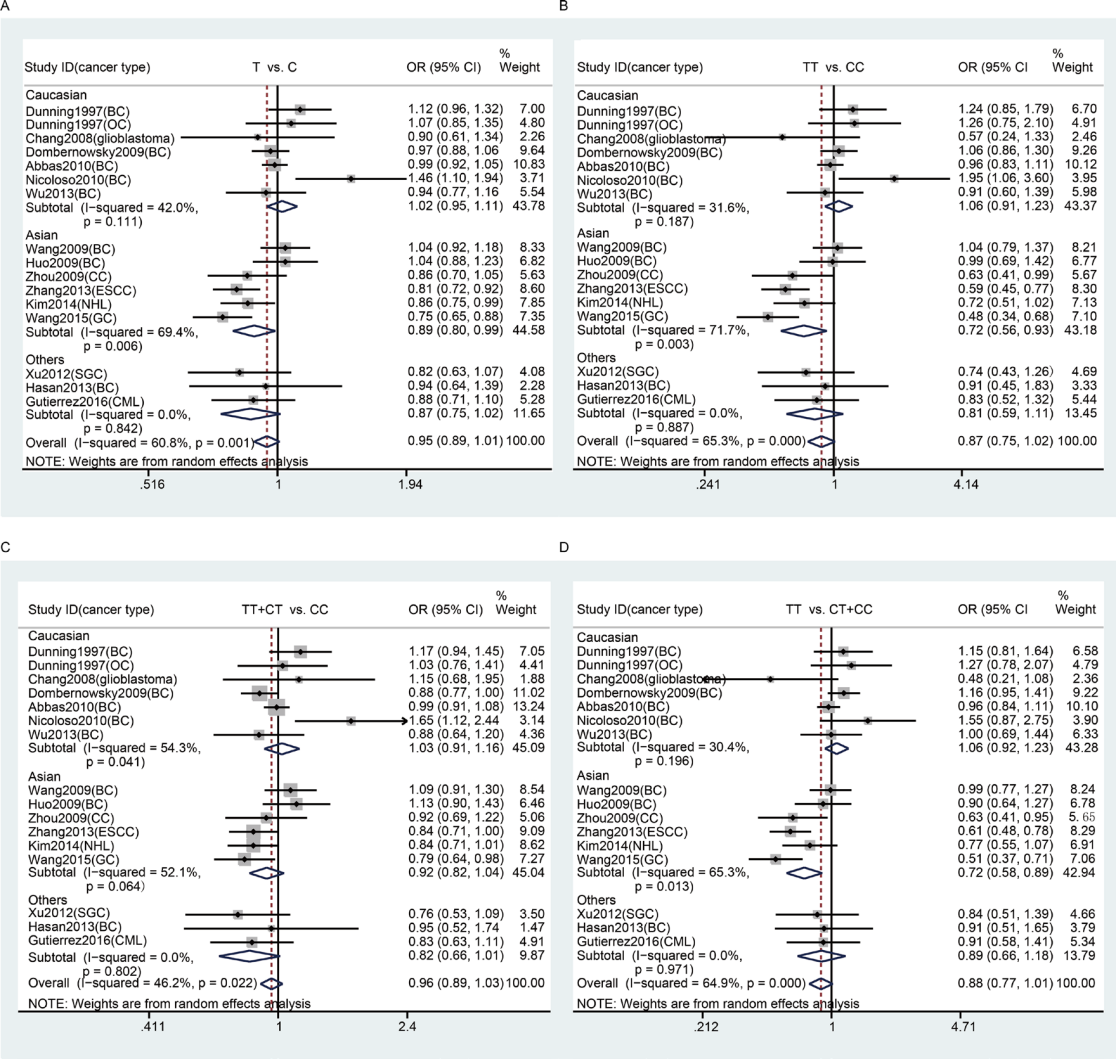
Stratification analyses by ethnicity between rs799917 polymorphism and cancer risk (**A**) allele model; (**B**) homozygous model; (**C**) dominant model; (**D**) recessive model. The squares and horizontal lines correspond to the study specific OR and 95% CI. The area of the squares reflects the weight. The diamond represents the summary OR and 95% CI. The random-effects model was used.

In the subgroup analyses by cancer type, we found that there was no significant association between rs799917 polymorphism and breast cancer risk either in the overall population or in the Caucasian, Asian, or other populations (Table 3 and Figure 2), which is consistent with previous reports [33]. There were eight studies included in our meta-analysis regarding rs799917 polymorphism and the risk of non-breast cancer. Among these, four studies were carried out in Asian populations, and four studies carried out in Caucasian or other populations. The studies on Asians demonstrated that rs799917 polymorphism could decrease the risk of cervical cancer, ESCC, gastric cancer, and NHL in one or more genetic models (for cervical cancer: TT vs. CC: OR, 0.64, 95% Cl, 0.41–0.99, *P* = 0.046; TT vs. CT + CC: OR, 0.63, 95%Cl, 0.41–0.95, *P* = 0.029; for ESCC: T vs. C: OR, 0.81, 95% Cl, 0.72–0.92, *P* = 0.001; TT vs. CC: OR, 0.59, 95% Cl, 0.45–0.77, *P* < 0.001; TT + CT vs. CC: OR, 0.84, 95% Cl, 0.71–1.00, *P* = 0.045; TT vs. CT + CC: OR, 0.61, 95% Cl, 0.48–0.78, *P* < 0.001; for gastric cancer: T vs. C: OR, 0.76, 95% Cl , 0.65–0.88, *P* < 0.001; TT vs. CC: OR, 0.48, 95% Cl, 0.34–0.68, *P* < 0.001; TT + CT vs. CC: OR, 0.79, 95% Cl, 0.64–0.98, *P* = 0.030; TT vs. CT + CC: OR, 0.51, 95% Cl, 0.37–0.71, *P* < 0.001; for NHL: T vs. C: OR, 0.86, 95% Cl, 0.75–0.99, *P* = 0.032, Table 3 and Figure 2). The studies on Caucasians and other populations demonstrated that there was no significant association between rs799917 polymorphism and ovarian cancer, Glioblastoma, SGC and CML in any genetic models (Table 3 and Figure 2). *BRCA1* has been considered a risk-related gene for breast cancer, and the studies included in our meta-analysis reveal that the *BRCA1* rs799917 polymorphism plays an important role in the risk of non-breast cancer, especially in Asian populations. Due to the limited number of studies on each type of cancer, the conclusions drawn from these studies should be interpreted with caution. We require more studies on the association between rs799917 polymorphism and risk of these types of cancer to verify these conclusions, and the biological function of the polymorphism should be investigated as well.

No significant association was observed between rs799917 polymorphism and cancer risk in subgroup analyses based on control source and quality score (Table 3).

### Meta-analysis of rs1799950, rs1799966, and rs16941

The main results of the meta-analysis for rs1799950, rs1799966, and rs16941 polymorphisms are shown in Table 4.

**Table 4:** Meta-analysis of the association between rs1799950, rs1799966, and rs16941 polymorphisms and cancer risk

Subgroup	No.	G vs. A			GG vs. AA			AG vs. AA			GG+AG vs. AA			GG vs. AG+AA		
		OR (95%Cl)	PZ	Ph	OR (95%Cl)	PZ	Ph	OR (95%Cl)	PZ	Ph	OR (95%Cl)	PZ	Ph	OR (95%Cl)	PZ	Ph
rs1799950																
Overall	7	0.93 (0.81–1.06)^∗^	0.257	0.062	0.44 (0.28–0.69)	<0.001	0.300	1.00 (0.92–1.09)	0.941	0.140	0.96 (0.84–1.09)^∗^	0.535	0.095	0.44 (0.28–0.69)	<0.001	0.308
					(excluded Xu)									(excluded Xu)		
Ethnicity																
Caucasian	6	0.94 (0.86–1.01)	0.105	0.101	0.44 (0.28–0.69)	<0.001	0.300	0.99 (0.91–1.08)	0.873	0.190	0.96 (0.88–1.05)	0.377	0.144	0.44 (0.28–0.69)	<0.001	0.308
Mix	1	1.44 (0.88–2.36)	0.152	----	----	----	----	1.48 (0.88–2.48)	0.138	----	1.48 (0.88–2.48)	0.138	----	----	----	----
Cancer type																
Breast cancer	5	0.89 (0.78–1.02)^∗^	0.088	0.072	0.46 (0.29–0.72)	0.001	0.233	0.98 (0.90–1.07)	0.675	0.231	0.95 (0.87–1.04)	0.280	0.135	0.46 (0.29–0.72)	0.001	0.244
other cancers	2	1.22 (0.89–1.67)	0.216	0.411	0.19 (0.01–3. 30)	0.253	----	1.39 (1.00–1.94)	0.052	0.767	1.32 (0.95–1.83)	0.103	0.574	0.18 (0.01–3.18)	0.242	----
					(excluded Xu)									(excluded Xu)		
rs1799966																
Overall	6	0.89 (0.78–1.02)^∗^	0.091	0.001	0.86 (0.69–1.09)^∗^	0.207	0.038	0.87 (0.73–1.03)^∗^	0.112	0.005	0.86 (0.72–1.02)^∗^	0.087	0.002	0.95 (0.86–1.06)	0.372	0.119
Ethnicity																
Caucasian	5	0.90 (0.78–1.04)^∗^	0.148	0.001	0.86 (0.67–1.11)^∗^	0.252	0.020	0.89 (0.73–1.07)^∗^	0.206	0.003	0.87 (0.72–1.06)^∗^	0.161	0.001	0.92 (0.75–1.14)^∗^	0.445	0.068
Mix	1	0.85 (0.64–1.12)	0.239	----	0.82 (0.43–1.56)	0.542	----	0.76 (0.52–1.11)	0.159	----	0.77 (0.54–1.11)	0.157	----	0.93 (0.50–1.73)	0.818	----
Cancer type																
Breast cancer	4	0.90 (0.76–1.05)^∗^	0.180	<0.001	0.89 (0.68–1.16)^∗^	0.382	0.016	0.85 (0.70–1.04)^∗^	0.114	0.002	0.85 (0.69–1.05)^∗^	0.127	<0.001	0.96 (0.79–1.17)^∗^	0.665	0.093
Other cancers	2	0.86 (0.69–1.08)	0.200	0.825	0.72 (0.43–1.21)	0.215	0.499	0.91 (0.66–1.24)	0.533	0.109	0.86 (0.64–1.16)	0.281	0.328	0.74 (0.45–1.23)	0.251	0.240
rs16941																
Overall	6	1.05 (0.99–1.12)	0.130	0.147	1.14 (0.99–1.31)	0.064	0.271	1.03 (0.88–1.19)^∗^	0.734	0.055	1.03 (0.90–1.18)^∗^	0.642	0.082	1.14 (1.00–1.31)	0.052	0.257
Ethnicity																
Caucasian	5	1.06 (1.00–1.13)	0.059	0.293	1.17 (1.01–1.35)	0.033	0.346	1.06 (0.91–1.24)^∗^	0.444	0.072	1.05 (0.97–1.14)	0.233	0.150	1.17 (1.01–1.33)	0.033	0.241
Mix	1	0.82 (0.62–1.08)	0.158	----	0.74 (0.39–1.40)		----	0.76 (0.52–1.12)	0.162	----	0.76 (0.53–1.09)	0.130	----	0.83 (0.45–1.55)	0.565	----
Cancer type																
Breast cancer	3	1.01 (0.93–1.10)	0.820	0.772	1.09 (0.90–1.31)	0.376	0.905	0.95 (0.84–1.06)	0.356	0.143	0.97 (0.87–1.09)	0.634	0.304	1.12 (0.94–1.34)	0.208	0.584
Other cancers	3	1.00 (0.78–1.27)^∗^	0.970	0.055	0.97 (0.57–1.64)^∗^	0.909	0.064	1.09 (0.96–1.23)	0.196	0.109	1.03 (0.77–1.37)^∗^	0.864	0.087	0.95 (0.58–1.57)^∗^	0.840	0.069

OR, odds ratio; 95% CI, 95% confidence interval; *P*OR, pool *P* value; *P*h, *P* value of heterogeneity test; *indicates that the OR, 95% Cl, and corresponding *P*OR were calculated based on the random-effects model; otherwise, the fixed-effects model was used.

**Figure 3:**
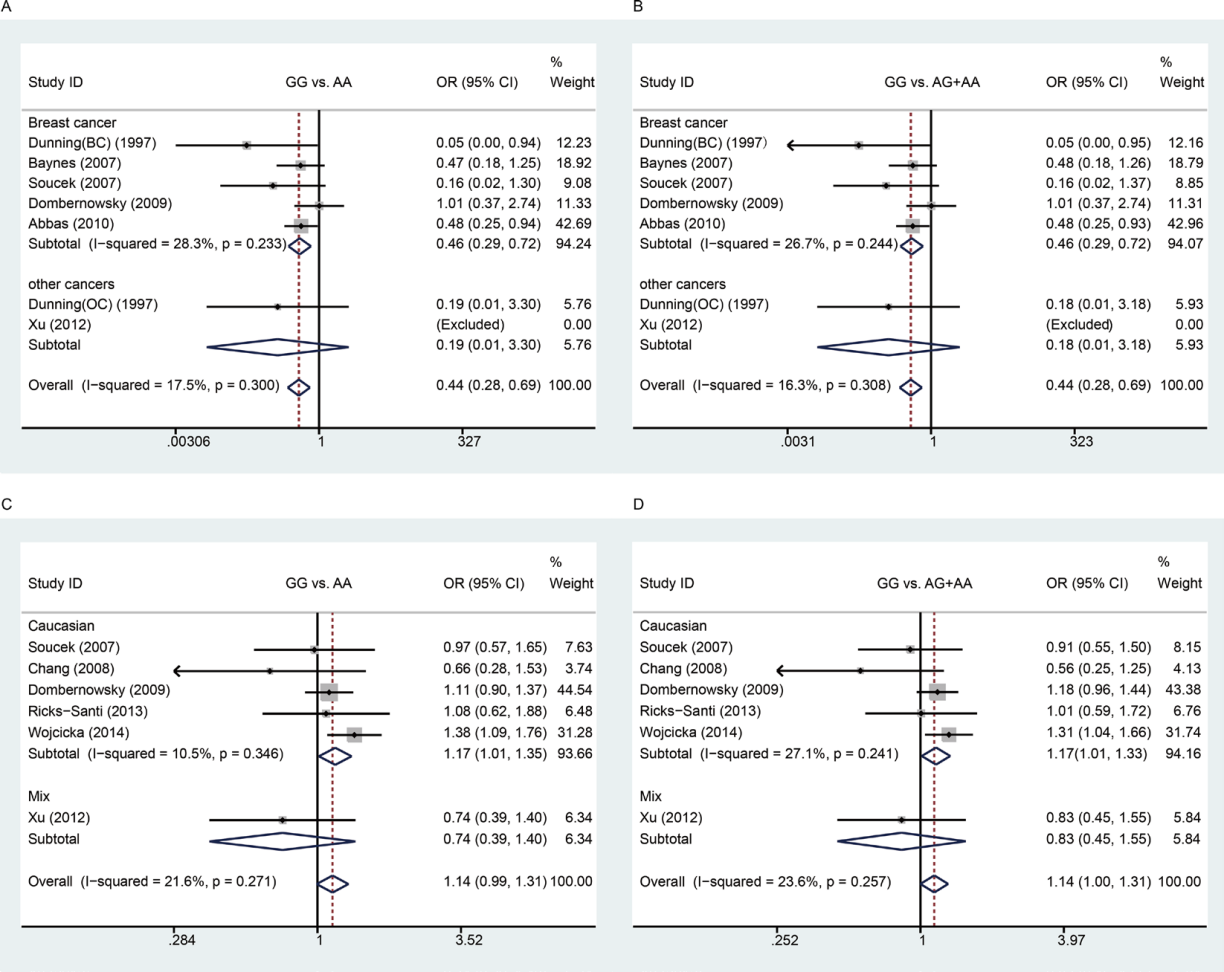
Meta-analysis between rs1799950 and rs16941 polymorphisms and cancer risk A and B: Stratification analyses by cancer type between rs1799950 polymorphism and cancer risk (**A**) homozygous model; (**B**) recessive model); C and D: Stratification analyses by ethnicity between rs16941 polymorphism and cancer risk (**C**) homozygous model; (**D**) recessive model). The squares and horizontal lines correspond to the study-specific OR and 95% CI. The area of the squares reflects the weight. The diamond represents the summary OR and 95% CI. The fixed-effects model was used.

There were seven studies with 7,997 cases and 13,951 controls examining rs1799950 polymorphism. We found that rs1799950 could decrease overall cancer risk (Table 4 and Figure 3). Furthermore, we found that rs1799950 could decrease cancer risk among Caucasian populations in the subgroup analyses based on ethnicity and rs1799950 could decrease breast cancer risk in the subgroup analyses based on cancer type. Because all of the studies on breast cancer were conducted using Caucasian populations, we suggest that rs1799950 can decrease breast cancer risk among Caucasian populations in the homozygote and recessive models (GG vs. AA: OR, 0.46, 95% Cl, 0.29–0.72, *P* = 0.001; GG vs. AG + AA: OR, 0.46, 95% Cl, 0.29–0.72, *P* = 0.001, Table 4 and Figure 3).

There were six studies with 5,371 cases and 10,910 controls examining rs1799966 polymorphism. We did not find any association between rs1799966 and cancer risk, either in the overall analysis or in subgroup analyses (Table 4).

There were six studies with 3,672 cases and 7,591 controls examining rs16941 polymorphism. We did not find association between rs16941 and overall cancer risk. However, in the subgroup analyses based on ethnicity, we found that rs16941 could increase cancer risk among Caucasian populations in the homozygote and recessive models (GG vs. AA: OR, 1.17, 95% Cl, 1.07–1.35, *P* = 0.033; GG vs. AG + AA: OR, 1.17, 95% Cl, 1.01–1.33, *P* = 0.033, Table 4 and Figure 3). No significant association was observed between rs16941 polymorphism and cancer risk in the subgroup analyses based on cancer type.

### Sensitivity analysis

**Figure 4:**
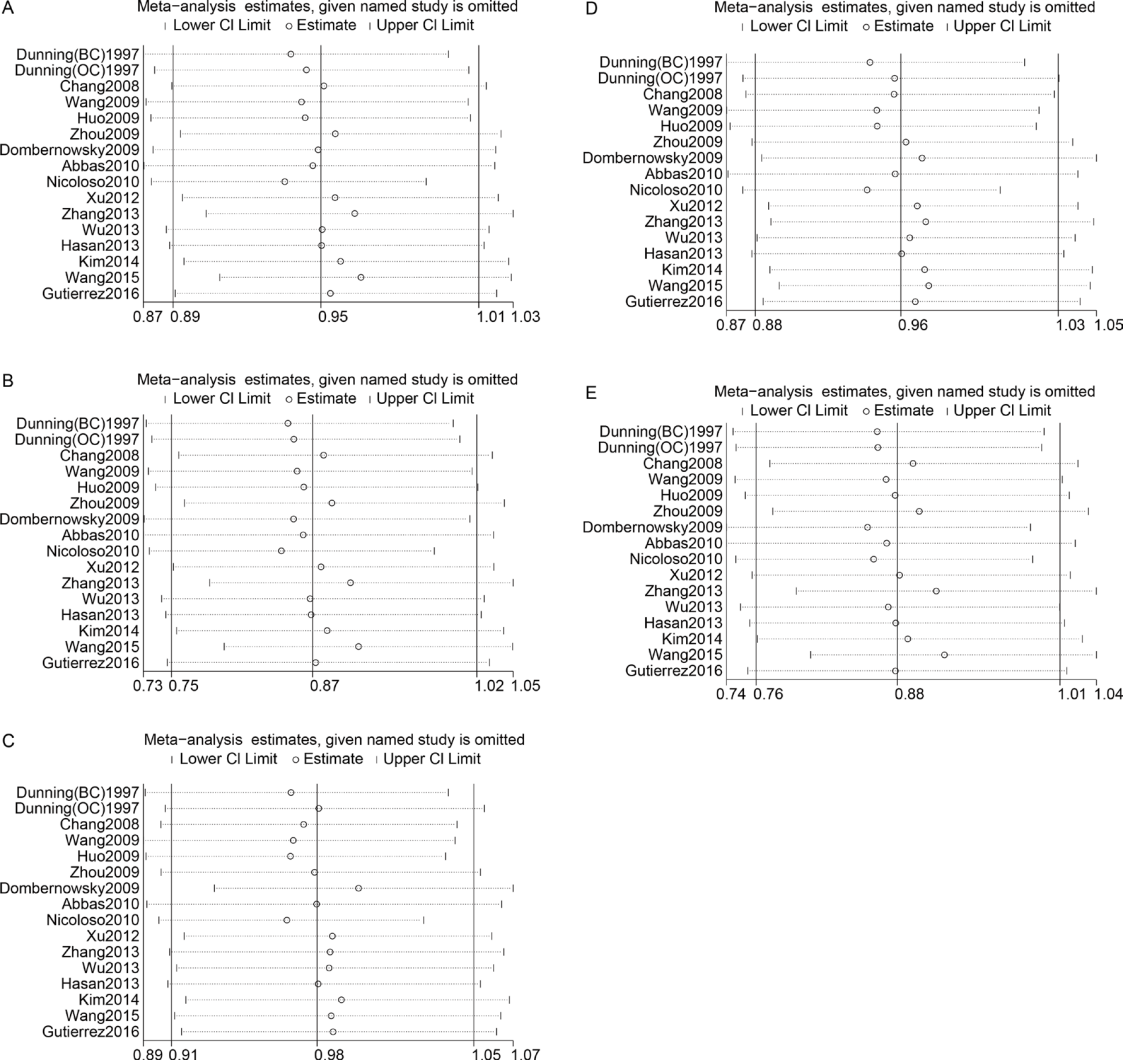
Sensitivity analyses between rs799917 polymorphism and cancer risk (**A**): allele model; (**B**): homozygous model; (**C**): heterozygous model; (**D**): dominant model; (**E**): recessive model. The random-effects model was used.

We assessed sensitivity by omitting a single study each time. The sensitivity analysis of rs799917 showed that our data were stable in the heterozygote and dominant genetic models but unstable in the allele, homozygote and recessive models (Figure 4 and Supplementary Table 2). In the allele and homozygote models, after omitting the study by Dunning et al. (BC) or Nicoloso et al., rs799917 was found to significantly decrease cancer risk, and in the recessive model, after omitting the study by Dunning et al. (OC), Dombernowsky et al., or Nicoloso et al., rs799917 was found to significantly decrease cancer risk.

No individual study showed a significant influence on the pooled ORs for rs1799950 and rs1799966 (Supplementary Table 3), indicating that our data were relatively stable for these polymorphisms

The sensitivity analysis of rs16941 showed that the results were stable in the allele, heterozygote, and dominant models but unstable in the homozygote and recessive models (Supplementary Table 3). In the homozygote model, after omitting the study by Chang et al. or Xu et al. rs16941 was shown to increase cancer risk, and in the homozygote model, after omitting the study by Soucek et al., Chang et al., Xu et al. or Ricks-Santi et al. rs16941 was shown to increase cancer risk.

### Publication bias

Both Begg’s test and Egger’s test were performed to assess the publication bias of the studies of these SNPs. Our data revealed that there was no obvious publication bias in any of the five models for rs799917, rs1799950 and rs1799966 (Table 5). There was publication bias in the recessive model for rs16941 (Egger’s test *P* = 0.015, Table 5).

**Table 5:** Publication bias analysis

Polymorphism	Genetic model	Egger’s test			Begg’s test
		t	95% Cl	*P*	*P*
rs799917	T vs. C	-0.08	-2.042–1.886	0.934	0.964
	TT vs. CC	-0.46	-2.574–1.659	0.650	0.822
	CT vs. CC	0.80	-0.915–1.998	0.439	0.444
	TT + CT vs. CC	0.39	-1.331–1.929	0.700	0.444
	TT vs. CT+CC	-0.70	-2.805–1.429	0.497	1.000
rs1799950	G vs. A	-0.41	-3.838–2.777	0.697	1.000
	GG vs. AA	-1.90	-3.500–0.657	0.130	0.260
	AG vs. AA	-0.03	-3.051–2.983	0.978	1.000
	GG+AG vs. AA	-0.23	-3.455–2.888	0.827	0.764
	GG vs. AG+AA	-1.87	-3.478–0.677	0.135	0.452
rs1799966	G vs. A	-1.09	-5.522–2.416	0.338	0.260
	GG vs. AA	-1.04	-4.217–1.911	0.355	0.452
	AG vs. AA	-0.84	-5.071–2.705	0.446	1.000
	GG+AG vs. AA	-0.98	-5.465–2.606	0.381	1.000
	GG vs. AG+AA	-0.88	-3.586–1.853	0.426	0.707
rs16941	G vs. A	-0.90	-4.186–2.131	0.417	0.452
	GG vs. AA	-2.21	-3.645–0.413	0.092	0.260
	AG vs. AA	0.50	-3.152–4.537	0.643	1.000
	GG+AG vs. AA	-0.04	-3.824–3.709	0.968	1.000
	GG vs. AG+AA	-4.12	-3.323- -0.647	0.015	0.06

The results of the sensitivity and publication bias analyses suggest that the number of studies included in our study was insufficient, especially for rs16941. Thus, the conclusions obtained by the current research should be interpreted with caution, and more studies are required to verify these conclusions in the future.

## DISCUSSION

Based on GLOBOCAN estimates, about 14.1 million new cancer cases and 8.2 million deaths occurred worldwide in 2012 [2]. Cancer is the result of a combination of genetic and environmental pressures; familial cancer is primarily based on hereditary factors, and environmental factors are the main cause of sporadic cancer [1]. Thus, genetic and environmental factors are both important in cancer research, including the study of association between SNPs and cancer.

*BRCA1* is a tumor suppressor gene, and its most important function is DNA repair. Thus far, many studies on *BRCA1* have been concerned with the risk of breast and ovarian cancer in *BRCA1* mutation carriers. There are several mutation types that lead to the protein inactivation of BRCA1, including frameshift, missense, nonsense, and splice mutations [13]. The mutation types tend to correspond with various populations and ethnicities [34]. Moreover, many studies have reported that *BRCA1* SNP is associated with cancer risk. For example, using data from Genome-Wide Association Study (GWAS), Li et al. investigated the associations between DNA repair pathway genes and the risk of ESCC and GC, finding *BRCA1* rs8176257 to be significantly associated with the risk of ESCC in a Han Chinese population [35]. In addition, Mullany et al. identified 327 SNPs that may play important roles in the regulation of miRNA expression based on the GWAS data. Two of these SNPs were significantly associated with colon cancer, one being *BRCA1* 8176318 [36]. To investigate the relationship between *BRCA1* SNPs and cancer risk, we conducted this meta-analysis.

Four *BRCA1* gene polymorphisms were evaluated: rs799917, rs1799950, rs1799966, and rs16941. The SNPs for the meta-analysis were selected using the following criteria: the SNP was reported to be associated with cancer risk in previous studies, the SNPs were non-synonymous, and the minor allele frequencies (MAFs) of the polymorphisms were greater than 1% in most of the populations from the 1000 Genomes Project Phase 3 (Supplementary Table 4). The linkage disequilibrium (LD) of the selected SNP is shown in Supplementary Figure 1.

rs799917 is a nonsynonymous SNP, and the rs799917 C > T variant leads to an amino acid change from proline to leucine at position 871 in the BRCA1 protein. This amino acid change may impact the interaction between miR-638 and *BRCA1* mRNA, leading to increased BRCA1 expression in rs799917 T allele carriers [23, 25]. In the meta-analysis, we revealed that rs799917 polymorphism could decrease the risk of several types of non-breast cancer. Thus, this study and previous studies have shown that rs799917 is not associated with the risk of BC [33]. Zhang et al. reported that there were significantly lower *BRCA1* mRNA levels among subjects with the rs799917 CC genotype than among those with the CT or TT genotypes in normal and cancerous esophageal tissues [25], which could explain why the risk of ESCC is lower among rs799917 T carriers.

Maia et al. performed an analysis of differential allelic expression (DAE) in *BRCA1* rs799917 heterozygous individuals (CT individuals) [37]. They found that in fresh blood B cells, the T allele was expressed at a higher level than the C allele in some heterozygous individuals (44%) and that the C allele was expressed at a higher level than the T allele in other heterozygous individuals (26%). Furthermore, they also found that the C allele was consistently expressed at a higher level than the T allele in breast tissue in all heterozygous individuals. This suggests that in the complex cellular environment, the DAE of *BRCA1* is not only influenced by miR-638 but also by other regulatory factors and that the regulatory factors for *BRCA1* rs799917 expression are different in fresh blood B cells and breast tissue.

rs1799950 is located in the region of exon 11 that binds Rad50, which is part of the DNA damage repair complex [38]. rs1799950 can be found in many families who are at a high-risk for prostate cancer [39]. Our meta-analysis revealed that rs1799950 could decrease the breast cancer risk among Caucasian populations. rs1799966 is located in the coding region of the COOH-terminal domain of BRCA1 (BRCT). The BRCT domain is an important signaling and protein-targeting motif in the DNA damage response system [40]. This suggests that rs1799966 may be important in the etiology of cancer. However, our results indicated that there was no observable association between rs1799966 and cancer risk, either in the overall or subgroup analysis. The presence of the rs16941 polymorphism has been reported to be significantly associated with the positive expression of BRCA1 protein [41]; however, we found that rs16941 could increase cancer risk among Caucasian populations. Therefore, we require more research to verify the results regarding rs16941 in the future.

There is nearly complete linkage disequilibrium between rs799917, rs1799966, and rs16941 in the most of the populations from the 1000 Genomes Project Phase 3 (Supplementary Figure 1). However, we ultimately chose to include these three SNPs instead of choosing only one because the studies on each SNP are very limited in number and the results for all the SNPs could complement one another and thus help to verify the conclusion. In our meta-analysis, we found that rs799917, rs1799966, and rs16941 were not associated with overall cancer risk. We suggest that among Asian populations rs799917 could decrease the risk of several types of cancer. However, for rs1799966 and rs16941, there are no studies about Asian populations included in the meta-analysis. We revealed that rs16941 could increase cancer risk among Caucasians but that rs799917 and rs1799966 could not. Taking into account the limited number of included studies and the fact that there was publication bias in the studies of rs16941, we require more evidence to verify the conclusions in the future.

Our meta-analysis has certain limitations. First, we only included studies in the PubMed, Embase and Web of Science databases and those written in English. This means that some studies published in other databases and written in other languages may have been ignored. Second, the meta-analysis contained only a few types of cancer. Therefore, in the future, we will require more data regarding various types of cancer to arrive at a more comprehensive conclusion. Third, the results for rs799917 and rs16941 are unstable in some genetic models, and there is publication bias in the studies on rs16941. Thus, the conclusions drawn from the current research should be interpreted with caution. Finally, because of the limited number of included studies, this meta-analysis did not consider gene-gene or gene-environment interactions.

In conclusion, our meta-analysis suggests that rs799917 polymorphism could decrease the risk of cervical cancer, ESCC, gastric cancer, and NHL among Asian populations. We also found that rs1799950 could decrease breast cancer risk among Caucasian populations and that rs16941 could increase the overall cancer risk among Caucasian populations. Considering the limited number of cancer types in and the sample size of this meta-analysis, more studies including various types of cancer are needed to investigate the association between *BRCA1* polymorphisms and cancer risk in the future.

## MATERIALS AND METHODS

### Search strategy

We searched the PubMed, Embase, and Web of Science databases for relevant studies about *BRCA1* polymorphisms and cancer risk that were published before July 31, 2017. The following keywords and terms were used for the search: “BRCA1” or “Breast cancer associated gene 1”; “rs799917”, “Pro871leu”, “rs1799950”, “Gln356Arg”, “rs1799966”, “Ser1613Gly”, “rs16941” or “Glu1038Gly”; “variant”, “mutation”, “polymorphism” or “SNP”; “cancer”, “carcinoma”, “neoplasm” or “tumor” and the combinations. Besides, the reference lists of identified studies were also screened carefully for potential articles.

### Inclusion and exclusion criteria

All studies included in the meta-analysis had to meet the following criteria: 1) the study investigated the association between *BRCA1* polymorphisms and cancer risk; 2) the study was a case-control or cohort study; 3) the study was written in English; 4) the study contained detailed genotyping data. We excluded comments, editorials, reviews, meta-analyses, and studies lacking sufficient data.

### Data extraction

Data were extracted from all eligible studies by two investigators (Gui-Ping Xu and Qing Zhao) working independently. Disagreements were resolved via discussion. The following information was extracted: name of first author, year of publication, country, cancer type, ethnicity, genotyping methods, source of controls, genotype distributions of cases and controls, and Hardy-Weinberg equilibrium (HWE) for controls. One of the studies only provides the percentage of each genotype, the complete data were obtained from the author [32].

### Quality score

The quality of the studies was independently evaluated by two reviewers (Ding Wang and Hua Zhou) based on a quality assessment scale (Supplementary Table 1). Any disagreement was resolved via discussion between the two reviewers. Total scores ranged from 0 (worst) to 15 (best) [42].

### Statistical analysis

All statistical analyses were performed using the STATA software (Version 12.0; Stata Corporation, College Station, TX, USA). ORs with corresponding 95% CIs were used to assess the strength of the association between *BRCA1* polymorphisms and cancer risk. The strength of the association was estimated in the allele genetic model (T vs. C), the homozygote model (TT vs. CC), the heterozygote model (CT vs. CC), the dominant model (TT + CT vs. CC), and the recessive model (TT vs. CT + CC). The significance of the pooled OR was determined with the Z-test, and *P*-values < 0.05 were considered statistically significant. The heterogeneity among studies was evaluated using a chi-square-based *Q* test and quantified by I^2^ [43]. If the result of the heterogeneity test was *P* > 0.1, which indicated that the heterogeneity among studies was not significant, ORs were pooled using the fixed-effects model (Mantel-Haenszel model) [44]. Otherwise, the random-effects model (DerSimonian and Laird model) was used [45]. Hardy-Weinberg equilibrium (HWE) in the control group for each study was examined using a chi-squared test and a *P* value less than 0.05 was considered as significant disequilibrium. Stratified analyses were performed based on ethnicity, cancer type, control source, and quality score. Sensitivity analyses were performed to assess the stability of the results by omitting one single study each time. Begg’s test and Egger’s testwere performed to examine potential publication bias [46, 47]. All *P* values were two-sided, and *P* < 0.05 was considered as statistically significant except for the *P* value of heterogeneity.

## SUPPLEMENTARY MATERIALS FIGURE AND TABLES







## Abbreviations

SNP: single nucleotide polymorphism
OR: odds ratio
CI: confidence interval
BC: breast cancer
OC: ovarian cancer
SGC: Salivary Gland Carcinoma
ESCC: esophageal squamous cell carcinoma
NHL: Non-Hodgkin Lymphoma
PTC: Papillary Thyroid Carcinoma
CML: chronic myeloid leukemia
MAF: minor allele frequency
GWAS: genome-wide association study
HWE: Hardy-Weinberg equilibrium
DAE: differential allelic expression
ASOs: allele-specific oligonucleotides
PCR-PIRA: PCR primer introduced restriction analysis assay
PCR-RFLP: PCR restriction fragment length polymorphism
MALDI-TOF MS: matrix-assisted laser desorption/ionization time-of-flight mass spectrometry
PB: population-based
HB: hospital-based
FB: family-based
