# Supplementary material for: The association between BRCA1 gene polymorphism and cancer risk: a meta-analysis
Source: Oncotarget. 2018 Jan 6;9(9):8681–94. doi: 10.18632/oncotarget.24064 (PMC5823592; doi:10.18632/oncotarget.24064)
Supplement: Supplementary file 1 [file oncotarget-09-8681-s001.pdf]

The association between BRCA1 gene polymorphism and cancer risk: a meta-analysis

SUPPLEMENTARY MATERIALS

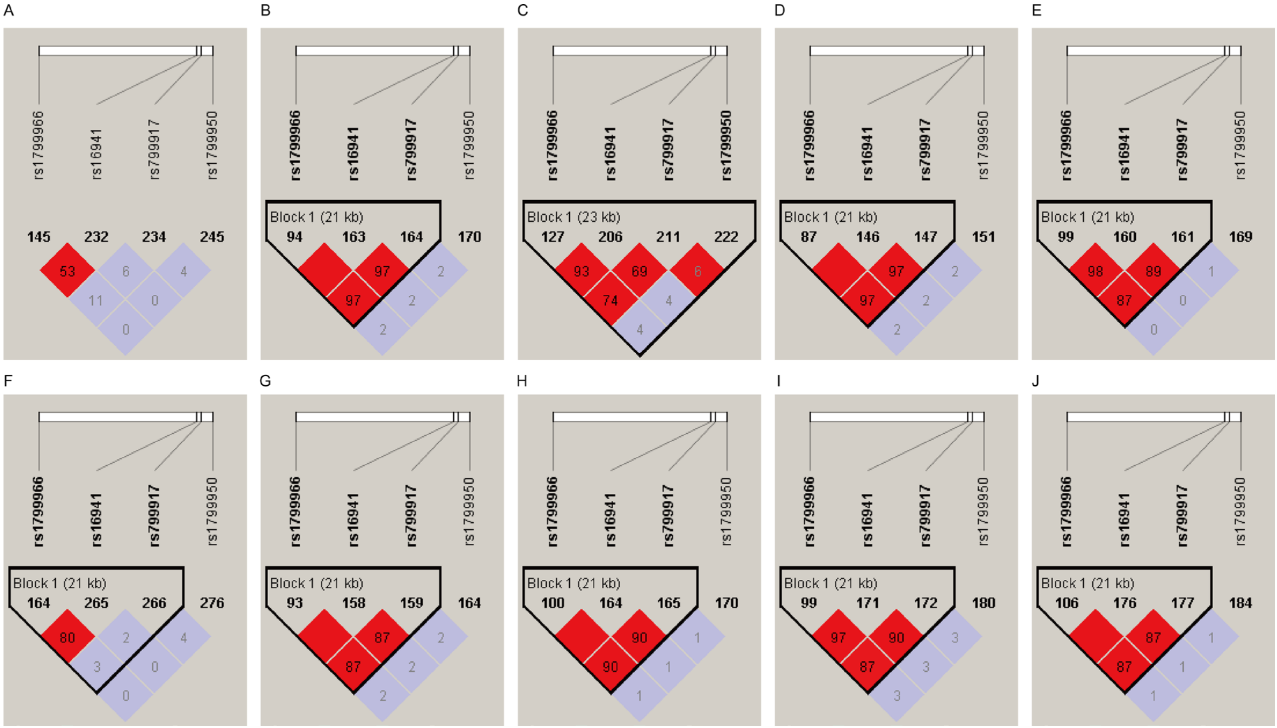

Supplementary Figure 1: Linkage disequilibrium analyses for BRCA1 polymorphisms in populations from the 1000 Genomes Project Phase 3. A. ASW; B. CEU; C. CLM; D. GBR; E. GIH; F. GWD; G. MXL; H.PEL; I. PJL; J. STU. ASW: Americans of African Ancestry in SW USA; CEU: Utah Residents with Northern and Western European Ancestry; CLM: Colombians from Medellin, Colombia; GBR: British in England and Scotland; GIH: Gujarati Indian from Houston, Texas; GWD: Gambian in Western Division in the Gambia; MXL: Mexican Ancestry in Los Angeles USA; PEL: Peruvians from Lima, Peru; PJL: Punjabi from Lahore, Pakistan.

Supplementary Table 1: Quality score assessment

rs799917	A	B	C	D	E	Score
Dunning[14](BC)	2	3	2	3	3	13
Dunning[14](OC)	2	3	2	3	2	12
Chang[17]	3	3	2	0	1	9
Wang[18]	2	3	3	3	3	14
Huo[19]	2	3	3	3	3	14
Zhou[20]	2	3	3	3	2	13
Dombernowsky[21]	2	3	2	3	3	13
Abbas[22]	2	3	2	3	3	13
Nicoloso[23]	0	0	3	3	1	7
Xu[24]	2	1	3	3	2	11
Zhang[25]	2	3	2	3	3	13
Wu[27]	3	1	3	3	2	12
Hasan[28]	2	1	3	0	1	7
Kim[29]	2	3	3	3	3	14
Wang[31]	2	3	2	3	3	13
Gutierrez[32]	2	2	3	3	2	12
**rs1799950**						
Dunning[14](BC)	2	3	2	0	3	10
Dunning[14](OC)	2	3	2	0	2	9
Baynes[15]	3	3	3	3	3	15
Soucek[16]	2	1	3	3	2	11
Dombernowsky[21]	2	3	2	3	3	13
Abbas[22]	2	3	2	0	3	10
Xu[24]	2	1	3	3	2	11
**rs1799966**						
Soucek[16]	2	1	3	3	2	11
Chang[17]	3	3	2	0	1	9
Dombernowsky[21]	2	3	2	3	3	13
Abbas[22]	2	3	2	3	3	13
Xu[24]	2	1	3	3	2	11
Wu[27]	3	1	3	3	2	12
**rs16941**						
Soucek[16]	2	1	3	3	2	11
Chang[17]	3	3	2	0	1	9
Dombernowsky[21]	2	3	2	3	3	13
Xu[24]	2	1	3	3	2	11
Ricks-Santi[26]	2	3	2	3	2	12
Wójcicka[30]	2	3	3	3	3	14

A-E represents the corresponding criterion in the table of scale for quality assessment criterion.

Supplementary Table 2: Sensitivity analyses for rs799917 polymorphism and cancer risk. See Supplementary_Table_2

Supplementary Table 3: Sensitivity analyses for rs1799950, rs1799966, and rs16941 polymorphisms and cancer risk. See Supplementary_Table_3

Supplementary Table 4: MAFs of rs799917, rs1799950, rs1799966, and rs16941 polymorphisms in the populations from the 1000 Genomes Project Phase 3

Populations	rs799917	rs1799966	rs16941	rs1799950
ACB	0.161	0.255	0.203	0005
ASW	0.262	0.246	0.148	0.016
BEB	0.477	0.5	0.5	0.006
CDX	0.5	0.5	0.5	N/A
CEU	0.364	0.359	0.359	0.04
CHB	0.35	0.35	0.35	N/A
CHS	0.376	0.381	0.376	N/A
CLM	0.484	0.441	0.426	0.053
ESN	0.056	0.172	0.126	N/A
FIN	0.359	0.359	0.359	0.081
GBR	0.324	0.319	0.319	0.044
GIH	0.49	0.476	0.481	0.01
GWD	0.088	0.257	0.217	0.004
IBS	0.36	0.374	0.374	0.079
ITU	0.441	0.485	0.485	N/A
JPT	0.264	0.264	0.264	N/A
KHV	0.379	0.379	0.379	N/A
LWK	0.116	0.227	0.116	N/A
MSL	0.112	0.235	0.159	N/A
MXL	0.414	0.383	0.383	0.031
PEL	0.4	0.376	0.376	0.024
PJL	0.49	0.469	0.464	0.036
PUR	0.38	0.308	0.288	0.067
STU	0.436	0.471	0.471	0.015
TSI	0.402	0.383	0.369	0.051
YRI	0.074	0.194	0.125	N/A

MAFs: minor allele frequencies; ACB: African Carribbeans in Barbados; ASW: Americans of African Ancestry in SW USA; BEB: Bengali from Bangladesh; CDX: Chinese Dai in Xi -shuangbanna, China; CEU: Utah Residents with Northern and Western European Ancestry; CHB: Han Chinese in Beijing, China; CHS: Southern Han Chinese; CLM: Colombians from Medellin, Colombia; ESN: Esan in Nigeria; FIN: Finnish in Finland; GBR: British in England and Scotland; GIH: Gujarati Indian from Houston, Texas; GWD: Gambian in Western Division in the Gambia; IBS: Iberian Population in Spain; ITU: Indian Telugu from the UK; JPT: Japanese in Tokyo, Japan; KHV: Kinh in Ho Chi Minh City, Vietnam; LWK: Luhya in Webuye, Kenya; MSL: Mende in Sierra Leone; MXL: Mexican Ancestry in Los Angeles USA; PEL: Peruvians from Lima, Peru; PJL: Punjabi from Lahore, Pakistan; PUR: Puerto Rican in Puerto Rico; STU: Sri Lankan Tamil from the UK; TSI: Toscani in Italia; YRI: Yoruba in Ibadan, Nigeria; N/A: MAF not available in these populations in 1000 Genomes.
