# Supplementary material for: The association between BRCA1 gene polymorphism and cancer risk: a meta-analysis
Source: Oncotarget. 2018 Jan 6;9(9):8681–94. doi: 10.18632/oncotarget.24064 (PMC5823592; doi:10.18632/oncotarget.24064)
Supplement: Supplementary file 2 [file oncotarget-09-8681-s002.docx]

**Table S2: Sensitivity analyses for rs799917 polymorphisms and cancer risk**

| **SNP** | **Comparison** | **Study omitted** | **Estimate** | **[95% Confident Interval]** | **Effect model** |
| --- | --- | --- | --- | --- | --- |
| **rs799917** | **T vs. C** | **Dunning(BC)** | **0.935** | **0.875-0.999** | Random |
|  |  | Dunning(OC) | 0.941 | 0.880-1.008 |  |
|  |  | Chang | 0.949 | 0.887-1.015 |  |
|  |  | Wang | 0.939 | 0.876-1.007 |  |
|  |  | Huo | 0.941 | 0.878-1.008 |  |
|  |  | Zhou | 0.953 | 0.890-1.021 |  |
|  |  | Dombernowsky | 0.946 | 0.879-1.019 |  |
|  |  | Abbas | 0.944 | 0.875-1.018 |  |
|  |  | **Nicoloso** | **0.933** | **0.878-0.990** |  |
|  |  | Xu | 0.953 | 0.891-1.020 |  |
|  |  | Zhang | 0.961 | 0.901-1.026 |  |
|  |  | Wu | 0.948 | 0.884-1.016 |  |
|  |  | Hasan | 0.948 | 0.886-1.014 |  |
|  |  | Kim | 0.955 | 0.892-1.024 |  |
|  |  | Wang | 0.964 | 0.906-1.025 |  |
|  |  | Gutierrez | 0.951 | 0.888-1.019 |  |
|  |  | Combined | 0.947 | 0.887-1.012 |  |
|  | **TT vs. CC** | **Dunning(BC)** | **0.852** | **0.729-0.996** | Random |
|  |  | Dunning(OC) | 0.857 | 0.733-1.002 |  |
|  |  | Chang | 0.883 | 0.757-1.030 |  |
|  |  | Wang | 0.860 | 0.730-1.012 |  |
|  |  | Huo | 0.866 | 0.736-1.017 |  |
|  |  | Zhou | 0.890 | 0.762-1.040 |  |
|  |  | Dombernowsky | 0.857 | 0.727-1.010 |  |
|  |  | Abbas | 0.865 | 0.726-1.031 |  |
|  |  | **Nicoloso** | **0.846** | **0.731-0.979** |  |
|  |  | Xu | 0.881 | 0.752-1.031 |  |
|  |  | Zhang | 0.906 | 0.784-1.048 |  |
|  |  | Wu | 0.871 | 0.742-1.023 |  |
|  |  | Hasan | 0.872 | 0.745-1.020 |  |
|  |  | Kim | 0.886 | 0.755-1.040 |  |
|  |  | Wang | 0.913 | 0.796-1.048 |  |
|  |  | Gutierrez | 0.876 | 0.747-1.027 |  |
|  |  | Combined | 0.873 | 0.750-1.016 |  |
|  | **CT vs. CC** | Dunning(BC) | 0.965 | 0.895-1.039 | Random |
|  |  | Dunning(OC) | 0.978 | 0.905-1.056 |  |
|  |  | Chang | 0.971 | 0.903-1.043 |  |
|  |  | Wang | 0.966 | 0.895-1.042 |  |
|  |  | Huo | 0.964 | 0.896-1.038 |  |
|  |  | Zhou | 0.976 | 0.903-1.054 |  |
|  |  | Dombernowsky | 0.997 | 0.928-1.070 |  |
|  |  | Abbas | 0.977 | 0.896-1.064 |  |
|  |  | Nicoloso | 0.963 | 0.902-1.027 |  |
|  |  | Xu | 0.984 | 0.914-1.060 |  |
|  |  | Zhang | 0.983 | 0.907-1.065 |  |
|  |  | Wu | 0.983 | 0.910-1.061 |  |
|  |  | Hasan | 0.977 | 0.906-1.054 |  |
|  |  | Kim | 0.988 | 0.915-1.068 |  |
|  |  | Wang | 0.984 | 0.909-1.064 |  |
|  |  | Gutierrez | 0.984 | 0.913-1.062 |  |
|  |  | Combined | 0.977 | 0.908-1.051 |  |
|  | **TT+CT vs. CC** | Dunning(BC) | 0.940 | 0.871-1.015 | Random |
|  |  | Dunning(OC) | 0.952 | 0.879-1.032 |  |
|  |  | Chang | 0.952 | 0.880-1.029 |  |
|  |  | Wang | 0.944 | 0.871-1.022 |  |
|  |  | Huo | 0.944 | 0.873-1.021 |  |
|  |  | Zhou | 0.958 | 0.883-1.038 |  |
|  |  | Dombernowsky | 0.965 | 0.888-1.050 |  |
|  |  | Abbas | 0.952 | 0.872-1.041 |  |
|  |  | Nicoloso | 0.939 | 0.879-1.003 |  |
|  |  | Xu | 0.963 | 0.891-1.041 |  |
|  |  | Zhang | 0.967 | 0.893-1.048 |  |
|  |  | Wu | 0.960 | 0.886-1.039 |  |
|  |  | Hasan | 0.955 | 0.883-1.034 |  |
|  |  | Kim | 0.967 | 0.892-1.048 |  |
|  |  | Wang | 0.969 | 0.896-1.047 |  |
|  |  | Gutierrez | 0.962 | 0.889-1.042 |  |
|  |  | Combined | 0.955 | 0.885-1.031 |  |
|  | **TT vs. CT+CC** | Dunning(BC) | 0.864 | 0.746-1.001 | Random |
|  |  | **Dunning(OC)** | **0.865** | **0.748-0.999** |  |
|  |  | Chang | 0.894 | 0.776-1.029 |  |
|  |  | Wang | 0.871 | 0.747-1.016 |  |
|  |  | Huo | 0.879 | 0.756-1.022 |  |
|  |  | Zhou | 0.899 | 0.778-1.038 |  |
|  |  | **Dombernowsky** | **0.856** | **0.740-0.990** |  |
|  |  | Abbas | 0.872 | 0.740-1.027 |  |
|  |  | **Nicoloso** | **0.861** | **0.748-0.992** |  |
|  |  | Xu | 0.882 | 0.761-1.023 |  |
|  |  | Zhang | 0.913 | 0.798-1.044 |  |
|  |  | Wu | 0.873 | 0.752-1.014 |  |
|  |  | Hasan | 0.879 | 0.759-1.018 |  |
|  |  | Kim | 0.889 | 0.766-1.033 |  |
|  |  | Wang | 0.919 | 0.810-1.044 |  |
|  |  | Gutierrez | 0.879 | 0.758-1.020 |  |
|  |  | Combined | 0.881 | 0.765-1.014 |  |
