# Supplementary material for: The association between BRCA1 gene polymorphism and cancer risk: a meta-analysis
Source: Oncotarget. 2018 Jan 6;9(9):8681–94. doi: 10.18632/oncotarget.24064 (PMC5823592; doi:10.18632/oncotarget.24064)
Supplement: Supplementary file 3 [file oncotarget-09-8681-s003.docx]

**Table S3: Sensitivity analyses for rs1799950, rs1799966, and rs16941 polymorphisms and cancer risk**

| **SNP** | **Comparison** | **Study omitted** | **Estimate** | **[95% Confident Interval]** | **Effect model** |
| --- | --- | --- | --- | --- | --- |
| **rs1799950** | **G vs. A** | Dunning(BC) | 0.953 | 0.833-1.091 | Random |
|  |  | Dunning(OC) | 0.911 | 0.789-1.052 |  |
|  |  | Baynes | 0.944 | 0.806-1.107 |  |
|  |  | Soucek | 0.957 | 0.851-1.076 |  |
|  |  | Dombernowsky | 0.911 | 0.770-1.078 |  |
|  |  | Abbas | 0.900 | 0.761-1.065 |  |
|  |  | Xu | 0.906 | 0.799-1.027 |  |
|  |  | Combined | 0.926 | 0.812-1.057 |  |
|  | **GG vs. AA** | Dunning(BC) | 0.521 | 0.327-0.828 | Fixed |
|  |  | Dunning(OC) | 0.504 | 0.317-0.801 |  |
|  |  | Baynes | 0.496 | 0.295-0.835 |  |
|  |  | Soucek | 0.520 | 0.325-0.831 |  |
|  |  | Dombernowsky | 0.406 | 0.242-0.679 |  |
|  |  | Abbas | 0.498 | 0.266-0.934 |  |
|  |  | Xu | ---- | ---- |  |
|  |  | Combined | 0.491 | 0.311-0.777 |  |
|  | **AG vs. AA** | Dunning(BC) | 1.014 | 0.928-1.109 | Fixed |
|  |  | Dunning(OC) | 0.993 | 0.910-1.084 |  |
|  |  | Baynes | 1.038 | 0.943-1.143 |  |
|  |  | Soucek | 1.019 | 0.933-1.112 |  |
|  |  | Dombernowsky | 1.010 | 0.918-1.112 |  |
|  |  | Abbas | 0.952 | 0.849-1.067 |  |
|  |  | Xu | 0.994 | 0.911-1.084 |  |
|  |  | Combined | 1.004 | 0.922-1.095 |  |
|  | **GG+AG vs. AA** | Dunning[(BC) | 0.981 | 0.852-1.129 | Random |
|  |  | Dunning(OC) | 0.941 | 0.819-1.080 |  |
|  |  | Baynes | 0.985 | 0.845-1.148 |  |
|  |  | Soucek | 0.987 | 0.876-1.110 |  |
|  |  | Dombernowsky | 0.953 | 0.804-1.129 |  |
|  |  | Abbas | 0.931 | 0.790-1.098 |  |
|  |  | Xu | 0.940 | 0.830-1.063 |  |
|  |  | Combined | 0.959 | 0.841-1.094 |  |
|  | **GG vs. AG+AA** | Dunning[(BC) | 0.521 | 0.327-0.828 | Fixed |
|  |  | Dunning[(OC) | 0.505 | 0.317-0.802 |  |
|  |  | Baynes | 0.495 | 0.294-0.832 |  |
|  |  | Soucek | 0.519 | 0.324-0.829 |  |
|  |  | Dombernowsky | 0.406 | 0.242-0.679 |  |
|  |  | Abbas | 0.503 | 0.269-0.944 |  |
|  |  | Xu | ---- | ---- |  |
|  |  | Combined | 0.492 | 0.311-0.777 |  |
| **rs1799966** | **G vs. A** | Soucek | 0.971 | 0.922-1.022 | Random |
|  |  | Chang | 0.891 | 0.772-1.027 |  |
|  |  | Dombernowsky | 0.860 | 0.702-1.053 |  |
|  |  | Abbas | 0.860 | 0.698-1.060 |  |
|  |  | Xu | 0.898 | 0.775-1.039 |  |
|  |  | Wu | 0.862 | 0.742-1.002 |  |
|  |  | Combined | 0.892 | 0.782-1.018 |  |
|  | **GG vs. AA** | Soucek | 0.957 | 0.854-1.073 | Random |
|  |  | Chang | 0.885 | 0.699-1.121 |  |
|  |  | Dombernowsky | 0.788 | 0.559-1.110 |  |
|  |  | Abbas | 0.799 | 0.548-1.165 |  |
|  |  | Xu | 0.861 | 0.666-1.113 |  |
|  |  | Wu | 0.812 | 0.631-1.046 |  |
|  |  | Combined | 0.863 | 0.686-1.085 |  |
|  | **AG vs. AA** | Soucek | 0.959 | 0.891-1.031 | Random |
|  |  | Chang | 0.842 | 0.704-1.007 |  |
|  |  | Dombernowsky | 0.851 | 0.651-1.112 |  |
|  |  | Abbas | 0.834 | 0.646-1.076 |  |
|  |  | Xu | 0.885 | 0.732-1.069 |  |
|  |  | Wu | 0.851 | 0.697-1.041 |  |
|  |  | Combined | 0.869 | 0.732-1.033 |  |
|  | **GG+AG vs. AA** | Soucek | 0.958 | 0.894-1.026 | Random |
|  |  | Chang | 0.841 | 0.698-1.012 |  |
|  |  | Dombernowsky | 0.832 | 0.637-1.088 |  |
|  |  | Abbas | 0.824 | 0.633-1.071 |  |
|  |  | Xu | 0.873 | 0.721-1.056 |  |
|  |  | Wu | 0.832 | 0.682-1.016 |  |
|  |  | Combined | 0.860 | 0.723-1.022 |  |
|  | **GG vs. AG+AA** | Soucek | 0.977 | 0.877-1.089 | Fixed |
|  |  | Chang | 0.963 | 0.866-1.072 |  |
|  |  | Dombernowsky | 0.919 | 0.812-1.039 |  |
|  |  | Abbas | 0.972 | 0.824-1.147 |  |
|  |  | Xu | 0.954 | 0.857-1.063 |  |
|  |  | Wu | 0.939 | 0.842-1.047 |  |
|  |  | Combined | 0.954 | 0.858-1.060 |  |
| **rs16941** | **G vs. A** | Soucek | 1.050 | 0.985-1.120 | Fixed |
|  |  | Chang | 1.052 | 0.988-1.120 |  |
|  |  | Dombernowsky | 1.901 | 1.006-1.183 |  |
|  |  | Xu | 1.063 | 0.998-1.133 |  |
|  |  | Ricks-Santi | 1.047 | 0.981-1.117 |  |
|  |  | Wójcicka | 0.991 | 0.916-1.071 |  |
|  |  | Combined | 1.049 | 0.986-1.117 |  |
|  | **GG vs. AA** | Soucek | 1.155 | 0.999-1.335 | Fixed |
|  |  | **Chang** | **1.162** | **1.007-1.339** |  |
|  |  | Dombernowsky | 1.173 | 0.971-1.446 |  |
|  |  | **Xu** | **1.169** | **1.012-1.350** |  |
|  |  | Ricks-Santi | 1.148 | 0.993-1.327 |  |
|  |  | Wójcicka | 1.034 | 0.870-1.230 |  |
|  |  | Combined | 1.143 | 0.994-1.316 |  |
|  | **AG vs. AA** | Soucek | 1.011 | 0.851-1.200 | Random |
|  |  | Chang | 1.005 | 0.862-1.173 |  |
|  |  | Dombernowsky | 1.096 | 0.960-1.252 |  |
|  |  | Xu | 1.062 | 0.910-1.239 |  |
|  |  | Ricks-Santi | 1.008 | 0.847-1.200 |  |
|  |  | Wójcicka | 0.994 | 0.828-1.193 |  |
|  |  | Combined | 1.026 | 0.882-1.194 |  |
|  | **GG+AG vs. AA** | Soucek | 1.021 | 0.872-1.195 | Random |
|  |  | Chang | 1.023 | 0.883-1.186 |  |
|  |  | Dombernowsky | 1.095 | 0.958-1.251 |  |
|  |  | Xu | 1.066 | 0.939-1.210 |  |
|  |  | Ricks-Santi | 1.015 | 0.867-1.188 |  |
|  |  | Wójcicka | 0.972 | 0.856-1.103 |  |
|  |  | Combined | 1.033 | 0.902-1.182 |  |
|  | **GG vs. AG+AA** | **Soucek** | **1.165** | **1.014-1.340** | Fixed |
|  |  | **Chang** | **1.168** | **1.019-1.339** |  |
|  |  | Dombernowsky | 1.118 | 0.933-1.341 |  |
|  |  | **Xu** | **1.163** | **1.013-1.334** |  |
|  |  | **Ricks-Santi** | **1.155** | **1.005-1.326** |  |
|  |  | Wójcicka | 1.066 | 0.904-1.258 |  |
|  |  | **Combined** | **1.145** | **1.001-1.309** |  |
